# Solitary fibrous pleural tumor. A rare and challenging case

**DOI:** 10.1016/j.ijscr.2019.12.020

**Published:** 2019-12-19

**Authors:** Saulat hasnain Fatimi, Hina Inam, Farida Karim Chagan, Usama Khalid Choudry

**Affiliations:** a Department of Cardiothoracic Surgery, Aga Khan University Hospital, Pakistan; b Department of Peadiatrics, Sacred Heart Hospital, Florida, USA; c Department of General Surgery, Shifa International Hospital, Islamabad, Pakistan

**Keywords:** Solitary fibrous pleural tumor, En bloc resection, Benign lesion, Thoracic surgery

## Abstract

**Introduction:**

Solitary fibrous tumor of the pleura (SFTP) is a rare tumor originating from mesenchymal tissue underlying the mesothelial pleural layer with only a limited number of reported cases. Benign and malignant SFTP usually appear as a well-defined, homogeneous, and rounded mass on imaging. Complete en bloc is usually the treatment of choice.

**Case Presentation:**

44 years old gentleman presented with the unintentional weight loss and chest discomfort for 6 months. A CT scan of chest showed a 30 × 20 × 20 cm heterogeneously enhancing soft tissue mass involving the entire right hemithorax. PET scan showed a localized disease. Therefore, a complete right upper lobectomy and complete removal of mass was done, that resulted in complete expansion of the middle and lower lobe. The patient remains stable till date. Biopsy revealed a fibrous pleural tumor.

**Conclusion:**

Complete surgical resection of the tumor is usually sufficient, but there are reported cases with recurrence. Wedge resection for complete excision can be carried out for tumors arising from visceral pleura. Extra pleural excision can be done without chest wall resection in tumors arising from the parietal pleura.

## Highlights

• Solitary fibrous pleural tumors are rare.
• Majority are benign lesions.
• Complete en bloc resection is curative for localized disease.

## 1 Introduction

Solitary fibrous tumor of the pleura (SFTP) is a rare tumor originating from mesenchymal tissue underlying the mesothelial pleural layer with only a limited number of reported cases to date [[1], [2], [3]]. The majority of them are pedunculated masses with benign histologic features. About 12 % of SFTP, however, are malignant and eventually lead to death through local recurrence or metastatic disease [4]. The former usually remains asymptomatic and hence continues to grow to fairly large sizes, ultimately, leading to development of obstructive and compressive symptoms like dyspnea and cough [5]. Indeed, the tumor’s behavior is often unpredictable and does not always correlate with histologic findings [6].

Benign and malignant SFTP usually appear as a well-defined, homogeneous, and rounded mass on the initial chest radiograph [7]. Computed tomographic scan (CT) usually demonstrates a well-delineated, homogeneous, and occasionally lobulated mass of soft tissue attenuation. Although no specific CT features have been described for the diagnosis of SFTP, the tumors typically appear in contact with the pleural surface and show displacement or invasion of the surrounding structures [8]. Magnetic resonance imaging has limited use in the assessment of pleural disease [9].

Histologic examination of the tumor usually discloses cellular areas alternating with hyalinized and/or necrotic areas. Spindle shaped cells typically have minimal nuclear pleomorphism and rare or absent mitoses. Numerous thin walled vessels constitute an additional feature of large tumors [10].

Immunohistochemistry has been an extremely useful tool to differentiate SFTP from mesotheliomas and other sarcomas over the last few years. Indeed, SFTP by definition is vimentin positive and keratin negative. In addition, CD34 is positive in most benign and malignant SFTP, whereas it remains negative for most other pulmonary tumors [11].

Complete en bloc surgical resection is the preferred treatment of benign and malignant varieties of the tumor. The pedunculated tumors attached to the visceral pleura can be effectively treated with a wedge resection of lung [4]. Large sessile tumors can be difficult to resect because of extensive adhesions and may occasionally require a lobectomy or a pneumonectomy in order to achieve complete resection [12].

Benign SFTP has a high cure rate and an 8 % local recurrence rate that is usually amenable to curative re-excision. Malignant SFTP, especially the more common sessile type, has a 63 % recurrence rate even with complete resection [13]. The majority of patients with recurrent disease die of the tumor within 2 years. Nevertheless, the overall long-term cure rate for all patients is 88%–92 %.1(3). The following case report has been written as per the SCARE guidelines [21].

## 2 Case presentation

**Fig. 1:**
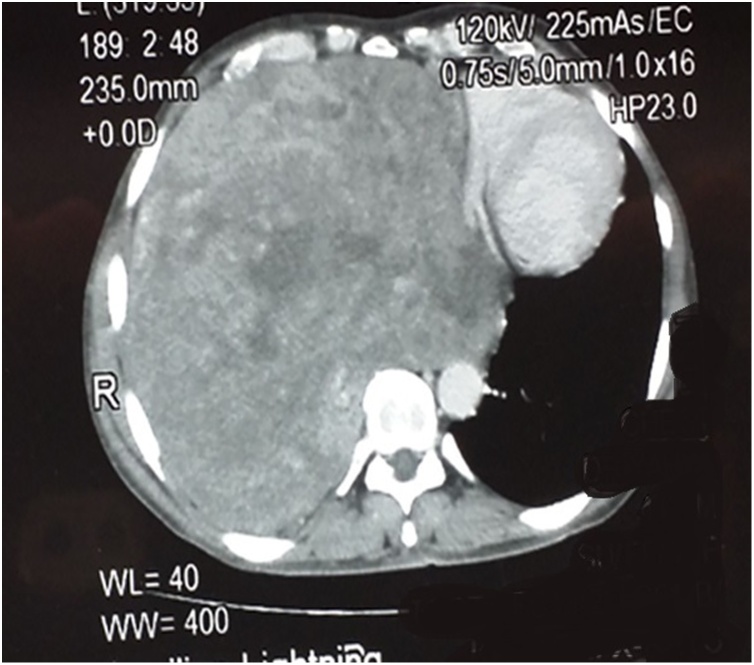
Showing CT imaging of chest demonstrating the mass.

**Fig. 2:**
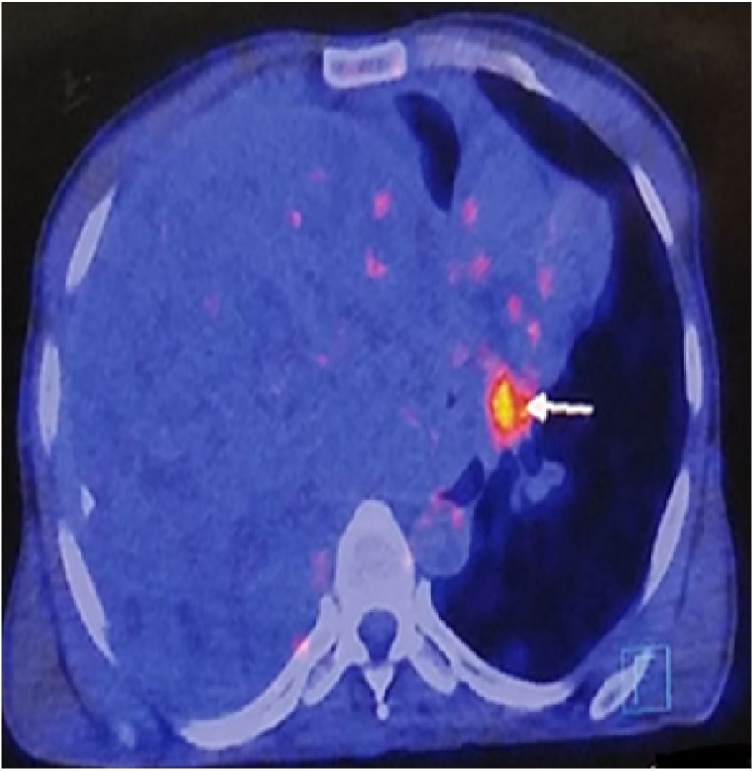
Showing PET imaging of chest.

44 years old gentleman presented in the cardiothoracic clinic with the unintentional weight loss of 7–8 kg for 1.5 years and chest discomfort for 6 months. On examination, he was a thin lean man with no obvious chest mass. Chest x-ray that showed a near complete opacification of right chest. A CT scan of chest was obtained which showed a 30 × 20 × 20 cm heterogeneously enhancing soft tissue mass involving the entire right hemithorax (Fig. 1). The mass was extending into the right hilum predominantly encasing the intermediate bronchus. There was evidence of completely collapsed right lung at the periphery. It was seen completely sheathing the right pulmonary artery and its branches and was observed to be inseparable from superior vena cava, right atrium, left atrium and superior and inferior pulmonary veins. Additionally, it enveloped 50 % of inferior vena cava and esophagus too. Posteriorly it involved the prevertebral fascia and 25 % of descending aorta. A CT guided biopsy was performed that showed spindle cell lesion with features favoring solitary fibrous tumor. This initial diagnosis was followed by a Positron Emission Tomography (PET) scan (Fig. 2) with the intent to see the spread of the disease, which indicated a localized disease.

However, owing to its rarity and rather unclear management guidelines, this case needed a multidisciplinary approach. Subsequently, oncology, onco-surgery and radiology experts were involved. With a general consensus, the patient was planned for a complete resection of the mass.

**Fig. 3:**
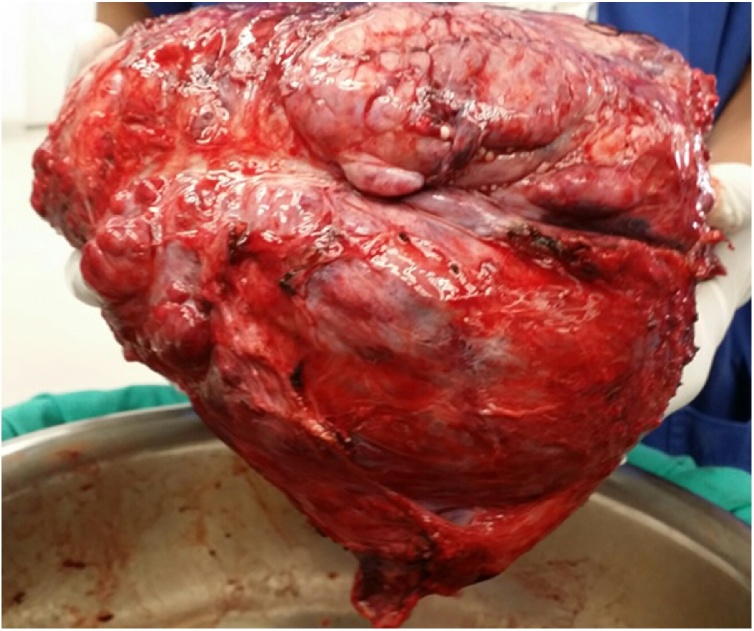
Showing the gross morphology of tumor.

An anterolateral thoracotomy was performed and intra-operatively, a giant highly vascular tumor was identified that occupied the entire right hemithorax compressing the right lung completely towards the periphery (Fig. 3). The tumor replaced by half of the right upper lobe of the lung. Therefore, a complete right upper lobectomy and complete removal of mass was achieved, that resulted in complete expansion of the middle and lower lobe. Postoperatively, the patient remained stable and was discharged on the fifth post-operative day.

The mass was sent for histopathology, which showed a neoplastic lesion composed of spindle to ovoid cells intermixed with ropy collagen bundles and thick walled staghorn vessels. These neoplastic spindle shaped cells were arranged in short intersecting fascicles. Sections examined from lung parenchyma showed dilated alveolar spaces with interstitial inflammation. Immunohistochemical stains were also performed resulting in CD34 and STAT-6 to be diffusely positive.

## 3 Discussion

SFTP are rare neoplasms that usually originate from the visceral with a global incidence of 5 % [14]. the disease has also been referred to as a localized mesothelioma, localized fibrous tumor, fibrous mesothelioma, or a pleural fibroma [15]. However, electron microscopy and immunohistochemistry has clarified that the tumor does not originate from the mesothelial layer but from the submesothelial, non-committed mesenchymal layer [16]. Thus, the various names used for this disease have become unified, and the disease is now referred to as solitary or localized fibrous tumors of the pleura.

Being more common in sixth - seventh decade of life, however, can present at any age with equal representation in both sexes. They are usually detected incidentally as small tumors, yet, being asymptomatic they can continue to achieve massive sizes and later on present with obstructive or compressive symptoms like cough, dyspnea [17]. These usually arise from visceral layer of the pleural cavity as similar to the reported case. Even though there are a few reported cases that are malignant but 80 % of patients have tumors that follow a benign course.

Preoperative radiologic and pathologic diagnosis is the guide to the suitable management approach. There are no specific radiologic features for the diagnosis of SFPT but CT scan is the most reliable. Heterogeneity may be observed with benign and malignant variants of SFTP because of myxoid degeneration, hemorrhage, or necrosis [7]. The role of CT guided biopsy has not been inconsistently and rather scarcely reported. Furthermore, surgical resection involves simultaneous diagnosis and treatment, preoperative biopsy should not always be considered.

Histopathologically, benign tumors are usually small in sizes with low cellularity, having a stalk and arising from the visceral pleura. While on the other hand huge benign tumors filling the whole hemi thorax have been reported, as was a case here [18]. Malignant forms do not have a stalk, originating from parietal, diaphragmatic or mediastinal pleura, being larger than 10 cm in sizes with increased cellularity and mitosis. Our patient had a localized lesion with some neoplastic features which was composed of spindle to ovoid cells intermixed with ropy collagen bundles and thick walled staghorn vessels.

SFPT lack distinctive histologic features, immunologic staining has frequently been employed to exclude other neoplasms in the differential diagnosis. Useful immunohistochemical prognostic markers of malignant SFTP are still being investigated. CD 34 has been widely been reported as positive marker that distinguishes the solitary fibrous tumor from most elements in the differential diagnosis [19] as was positive in our patient.

Although complete surgical resection of benign SFTP is the usual method of cure, occasional reports advise caution concerning its unpredictable clinical behavior such as its invasion of adjacent organs [16] or cardiac compression by the huge mass of benign SFTP [20]. The treatment of choice for benign SFTP is complete surgical resection. However, as far as malignant SFTP cases are concerned, there is no established systemic therapy, either preoperatively or postoperatively, despite the fact that malignant SFTP has shown distant metastasis [4]. This mandates the need for publication of this case report to further help the surgeons in deciding their management plan based on our experience.

## 4 Conclusion

Complete surgical resection of the tumor is usually sufficient, but there are reported cases with recurrence. Wedge resection for complete excision can be carried out for tumors arising from visceral pleura. Extra pleural excision can be done without chest wall resection in tumors arising from the parietal pleura.

## Sources of funding

None.

## Ethical approval

The ethical approval was exempted by the instituition.

## Consent

Written informed consent was obtained from the patient for publication of this case report and accompanying images. A copy of the written consent will be made available for review by the Editor-in-Chief of this journal on request"

## Author contribution

1. Saulat hasnain fatmi – DATA ANAYLSIS, INTERPRETATION, MANUSCRIPT DRAFTING.
2. Hina Inam – STUDY CONCEPT, STUDY DESIGN, DATA COLLECTION, MANUSCRIPT WRITING
3. Farida Karim – STUDY CONCEPT, DATA ANALYSIS, PROOF READING
4. Usama Khalid Choudry – CRITICAL REVIEW OF LITERATURE, DATA INTERPRETATION, proof reading

## Registration of research studies

Not acquired.

## Guarantor

Dr. Saulat hasnain fatmi Associate professor, Department of Cardiothoracic surgery. AGA KHAN University HOSPITAL

## Provenance and peer review

Not commissioned, externally peer-reviewed

## Declaration of Competing Interest

None.
